# Incidence of rapid increase of plasma sodium during liver transplantation and ITS effect on outcome

**DOI:** 10.1186/2197-425X-3-S1-A695

**Published:** 2015-10-01

**Authors:** NT Yoh, T Lai, G Wagener

**Affiliations:** Columbia University, Anesthesiology, New York, United States

## Introduction

Rapid correction of hyponatremia can cause pontine myelinolysis and should be corrected by less than 10 mM/L within 24 hours. Hepatic cirrhosis is often associated with hyponatremia due to fluid retention and ascites. During liver transplantation, sodium may increase because of fluid administration and blood transfusions. Little is known regarding the frequency of rapid sodium increases during liver transplantation and whether such rapid corrections carry detrimental effects.

## Objectives

To determine the incidence of rapid plasma sodium correction in liver transplants and their effect on outcome.

## Methods

We retrospectively analyzed data from 607 liver transplants at a single institution. Rapid correction of sodium was defined as an increase of serum sodium by more than 10 mM/L within 24 hours after the start of liver transplantation when compared to preoperative sodium levels. Primary endpoint was 90-day mortality or graft failure requiring re-transplantation.

## Results

**Figure 1:**
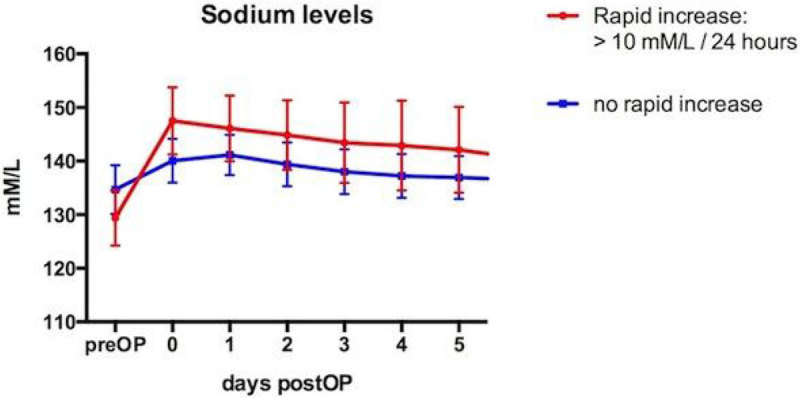
**Na over time-all patients.**

**Figure 2:**
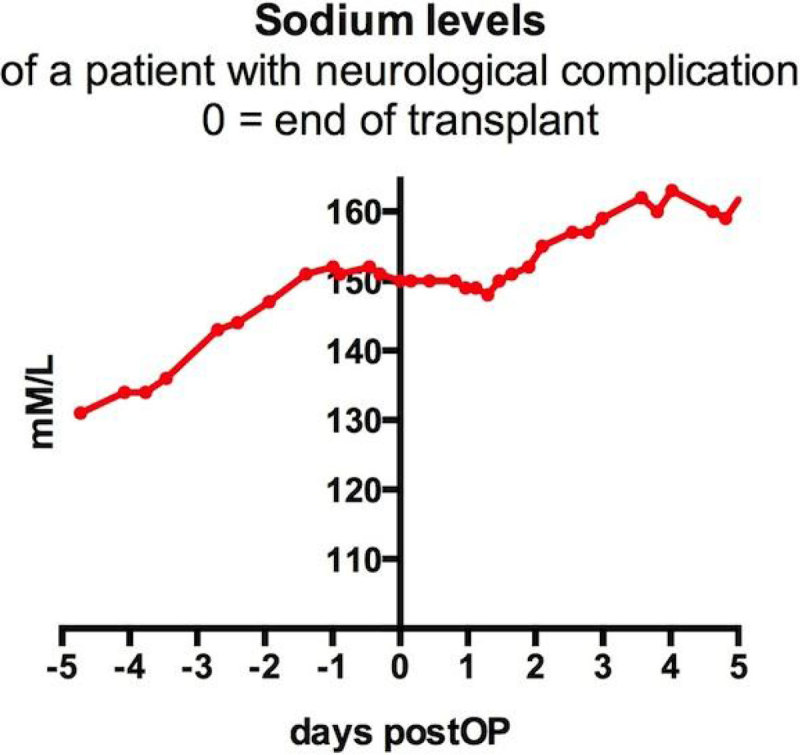
**Na case.**

Plasma sodium concentrations increased by more than 10 mM/L within 24 hours after liver transplantation in 39 of 607 patients (6.4%). These patients had lower preoperative sodium levels (129 +/-5.2 vs. 134 +/- 4.5 mM/L, p < 0.001) and higher MELD scores (33.9 +/- 7.1 vs. 20.4 vs. 10.2, p < 0.001). Sodium levels increased by 14.6 +/- 3.9; range 11-28 mM/L in the rapid increase group (figure 1). 39 patients died or required re-transplantation within 90 days. Even though the group with rapid Na increases had higher preoperative MELD scores there was no significant difference with regard to 90-day mortality/graft failure compared to patients with rapid increase and no rapid increase of sodium (4/39 - 10.3% vs. 35/568 or 6.2%, ns). One patient with a rapid perioperative increase of sodium (from 131 mM/L preoperative to 151 mM/L immediately after transplantation (figure 2)) had cerebral edema and severe cortical necrosis postoperatively and died 46 days after transplantation. However, this patient had acute decompensated hepatitis and severe mental status changes prior to transplantation.

## Conclusions

Perioperative rapid increases of sodium above what is recommended for correction of hyponatremia occurred in over 6% of all cases with only one case of severe neurological complication that was not consistent with pontine myelinolysis. Perioperative increases of plasma may be less dangerous than previously thought if it is not associated with abrupt changes of plasma osmolality. The neurological complication (cerebral edema, not pontine myelinolysis) of the patient described above are more likely attributable to her preoperative acute liver failure than rapid increase of sodium concentrations.

## References

[1] New England Journal of Medicine. 2000, 342 (21): 1581-1589. 10.1056/NEJM200005253422107. 10.1056/NEJM200005253422107 10824078

[2] Am J Med. 2013, 126 (10 Suppl 1): S1-42. Oct 10.1016/j.amjmed.2013.07.006 24074529

